# Differential expression patterns of genes associated with metabolisms, muscle growth and repair in *Pectoralis major* muscles of fast- and medium-growing chickens

**DOI:** 10.1371/journal.pone.0275160

**Published:** 2022-10-03

**Authors:** Yuwares Malila, Krittaporn V. Thanatsang, Pornnicha Sanpinit, Sopacha Arayamethakorn, Francesca Soglia, Martina Zappaterra, Martina Bordini, Federico Sirri, Wanilada Rungrassamee, Roberta Davoli, Massimiliano Petracci

**Affiliations:** 1 National Center for Genetic Engineering and Biotechnology (BIOTEC), Pathum Thani, Thailand; 2 Department of Agricultural and Food Sciences (DISTAL), Alma Mater Studiorum, University of Bologna, Cesena (FC), Italy; 3 Department of Agricultural and Food Sciences (DISTAL), Alma Mater Studiorum, University of Bologna, Bologna (BO), Italy; Tokat Gaziosmanpasa Universitesi, TURKEY

## Abstract

The aim of this study was to investigate the expression of genes related to muscle growth, hypoxia and oxidative stress responses, a multi-substrate serine/threonine-protein kinase (AMPK) and AMPK-related kinases, carbohydrate metabolism, satellite cells activities and fibro- adipogenic progenitors (FAPs) in fast-growing (FG) (n = 30) and medium-growing (MG) chickens (n = 30). *Pectoralis major* muscles were collected at 7d, 14d, 21d, 28d, 35d and 42d of age. According to their macroscopic features, the samples from FG up to 21d of age were classified as unaffected, while all samples collected at an older age exhibited macroscopic features ascribable to white striping and/or wooden breast abnormalities. In contrast, MG samples did not show any feature associated to muscle disorders. The absolute transcript abundance of 33 target genes was examined by droplet digital polymerase chain reaction. The results showed differential gene expression profiles between FG and MG chickens at different ages. While most genes remained unchanged in MG chickens, the expression patterns of several genes in FG were significantly affected by age. Genes encoding alpha 1, alpha 2, beta 2 and gamma 3 isoforms of AMPK, as well as AMPK-related kinases, were identified as differentially expressed between the two strains. The results support the hypothesis of oxidative stress-induced muscle damage with metabolic alterations in FG chickens. An increased expression of *ANXA2*, *DES*, *LITAF*, *MMP14*, *MYF5* and *TGFB1* was observed in FG strain. The results suggest the occurrence of dysregulation of FAP proliferation and differentiation occurring during muscle repair. FAPs could play an important role in defining the proliferation of connective tissue (fibrosis) and deposition of intermuscular adipose tissue which represents distinctive traits of muscle abnormalities. Overall, these findings demonstrate that dysregulated molecular processes associated with myopathic lesions in chickens are strongly influenced by growth rate, and, to some extent, by age.

## Introduction

Over the past decades, *Pectoralis major* (*P*. *major*) muscles of commercial broilers and turkeys belonging to fast-growing genotypes/hybrids have been affected by growth-related myopathies, known as White Striping (WS), Wooden Breast (WB), and Spaghetti Meat (SM). Such issue has raised the attention of poultry experts as it is associated with reduced technological properties of the breast meat and causes considerable economic loss for the poultry industry [1].

An investigation regarding molecular mechanisms associated with development of the abnormalities has been extensively conducted within the last years with an attempt to obtain a better understanding as well as the underlying etiology of the myopathies. Although the actual cause remained unclear, a collective evidence has pointed to a link between breeding selection for growth efficiency and the occurrence of myopathic disorders. As reviewed by Velleman [2], selection in meat-type birds focusing mainly on growth rate, feed conversion and muscle mass has affected muscle structure and muscle metabolism in the breast muscle. These changes have increased myodegeneration and necrosis but have limited muscle repair mediated by satellite cell.

In particular, research has shown that limited oxygenation due to reduced vascularization in hypertrophic breasts can be considered as a triggering factor for changes in biological pathways, contributing to the onset of these myopathic disorders [3, 4]. Among those, differential expression of genes involved in hypoxia, oxidative stress response and carbohydrate metabolism was identified in pectoralis major muscle of commercial broilers affected with growth-related myopathies, i.e., WS and WB, compared to the muscle samples of normal birds [5, 6]. Using the metabolomic technique, Abasht et al. [7] reported deviated glucose utilization, increased protein levels and altered redox homeostasis in the WB muscle. Moreover, signs of muscle regeneration have been occasionally observed in WS [8–10], but the number of regenerated muscles was significantly increased in WB compared to normal muscles [11–13].

In a recent study by Soglia et al. [14], an increase in vimentin (VIM) and desmin (DES) was found at both transcriptional and protein levels in WB muscles compared to normal counterparts, supporting an intensive regenerative process in the affected muscles. In addition, Soglia et al. [14] reported that abundance levels of VIM and DES were greater in the muscles of fast-growing chickens than in those of slow-growing birds. In this context, it has been hypothesized that the disrupted muscle repair processes in WS- and WB-affected breast muscle could be partly due to an excessive activation of satellite cells, leading to their exhaustion [15]. In myopathic muscles, accumulation of extracellular matrix (ECM) components was observed [16]. Moreover, Bordini et al. [17] recently reported that several genes encoding ECM components are the most interconnected nodes in gene network associated with the development of WS and WB abnormalities, suggesting that altered ECM might somehow activate the cascade of biological processes leading to the onset of the myopathies.

Given that 5’ AMP-activated protein kinase (AMPK), a multi-substrate serine/threonine-protein kinase, is widely known as an intracellular energy sensor, its role in development of growth-related myopathies has rarely been investigated. AMPK plays an essential role in maintaining cellular energy balance [18]. AMPK is activated under various stress conditions, such as oxidative stress [19] and when the cellular ATP decreases [20]. In addition, AMPK plays a key role in regulating skeletal muscle growth, development, and repair [21]. Based on the study of Rajakylä et al. [22], inhibition of either AMPK or Ca^2+^/calmodulin-dependent kinase kinase β (CaMKK β), encoded by *CAMKK2*, resulted in elevation of VIM in epithelial cells. Hence, AMPK, CaMKK and the others in CaMKKβ/AMPK pathway might be involved in development of growth-related myopathies.

As reviewed by Yang and Hu [23], under normal condition, efficient muscle regeneration depends greatly on a hierarchical program engaged by satellite cells and a tightly regulated participation of other cell types, including immune cells, vessel-associated cells, and mesenchymal cells. Upon the injury, the infiltrated pro-inflammatory macrophages secrete pro-inflammatory cytokines, particularly TNF-α, which attract other immune cells to the injury site and stimulate satellite cells proliferation [23]. Afterwards, the pro-inflammatory phenotype is converted to an anti-inflammatory condition, and this transition is regulated by AMPKα [24]. The pro-inflammatory macrophages are then converted to anti-inflammatory macrophages, which secrete transforming growth factor type beta (TGF- β) to initiate satellite cell differentiation and thus muscle repair [25]. Apart from muscle satellite cells, fibro-adipogenic progenitors (FAPs), a population of mesenchymal cells located in the interstitial area of the skeletal muscle, become activated, expand and differentiate into several mesenchymal lineages, including activated fibroblasts, adipocytes and bone-like cells, to provide transient favorable support for satellite cell differentiation [26, 27]. FAPs are reciprocally regulated by satellite cells in the early phase and later undergo TNF-α-induced apoptosis to remove excessively expanded FAPs and then return to basal levels [28]. Dysregulations in FAPs removal lead to an accumulation of FAPs, further differentiating into connective tissues, ultimately resulting in an extensive proliferation of collagen (or fibrosis) in the muscle [29]. Such phenomenon has been frequently observed under pathological conditions [30–33], demonstrating a positive correlation between fibrosis severity, FAP expansion and TGF-β gene expression level. In addition, a regulatory role of TGF-β signaling on the expression and function of platelet-derived growth factor receptor alpha subunit (PDGFRα), a receptor tyrosine kinase (RTK) expressed on the cell surface of FAPs, was identified. Overactivated TGF-β signaling reduced PDGFRα expression in FAPs, and promotes myofibroblast differentiation of FAPs but inhibits their adipogenicity, causing fibrosis. In chicken breast muscle affected with WB abnormality, PDGFRα transcript abundance was increased compared with that of non-WB counterparts [34, 35]. However, little is known about the role of FAPs either in chicken *P*. *major* muscle during post-hatch growth or growth-related myopathies.

Considering the limited knowledge about expression levels of AMPK, AMPK-related genes, and FAPs associated with growth-related myopathies in chickens, the aim of this study was to evaluate the absolute expression of those genes in *P*. *major* muscle during post-hatch muscle development (from 7 to 42 days of age) in fast-growing (FG) and medium-growing (MG) chickens. The presence of myopathic characteristics of the muscles was also monitored macroscopically and histologically. The selected genes included those associated with muscle growth, oxidative stress response, energy metabolism, and muscle regeneration previously identified in skeletal muscle of commercial broilers affected with WS and WB abnormalities. The findings could lead to a better understanding of the onset of growth-related myopathies in commercial broilers.

## Materials and methods

### Animal and sample collection

The study was approved by the Ethical Committee of the University of Bologna (ID: 1194/2021).

A total of 100 one-day-old male chicks (70 fast-growing, FG; 30 medium-growing, MG) were vaccinated at the hatchery (coccidiosis, infectious bronchitis, Marek’s, New-castle, and Gumboro disease) and transferred to an environmentally controlled poultry facility. As for the genetic lines, FG birds belong to Ross 308 hybrids whereas the MG ones belong to a female JA57 x RedBro male line.

The broilers were raised and slaughtered under commercial conditions in accordance with current legislation (EU legislation, Directive 2007/43/EC and Directive 2010/63/EU). In the experimental poultry house, chicks were randomly allotted in 6 m^2^ pens and, in compliance with the legislation in force, stocking density was defined to reach a maximum of 33 kg/m^2^. Each pen was provided with pan feeders (2 cm of front space/bird) and an independent drinking system (1 nipple/5 birds). Chopped straw (2 kg/m^2^) was utilized as litter. As for the feeding conditions, the same commercial corn-wheat-soybean basal diet was provided to both genotypes according to a 3-phase feeding program: starter (0–14 d), grower (15–28 d), and finisher (29-end). During the whole period, animals were fed *ad libitum*. An artificial photoperiod of 23 h light and 1 h dark was employed during the first 7 and last 3 days of the trial, whereas 18 h light and 6 h dark were used for the remaining time. The environmental temperature was settled based on the birds’ age and following breeding company instructions.

*P*. *major* muscles were collected from fast-growing (FG) and medium-growing (MG) chickens at the age of 7d, 14d, 21d, 28d, 35d, and 42d. In detail, for each sampling time, the superficial section of the cranial portion of each *P*. *major* muscle has been collected from 10 FG and 5 MG broilers at each age, following the sampling procedure reported by Soglia et al. [14]. All collected samples had been macroscopically evaluated to classify them as abnormal (i.e., affected by growth-related myopathies, namely WS and/or WB) or normal (i.e., not exhibiting any macroscopic feature associated to the abovementioned disorders). Macroscopic evaluation was performed by two well-trained operators. In particular, chicken breast muscles were evaluated by visual examination and palpation and scored following the criteria recently reviewed by Petracci et al. [4]. For instance, *P*. *major* muscles characterized by extreme firmness and very thick stripes (> 1 mm) with extensive coverage over the breast surface have been classified as “severely affected” by muscular abnormalities (ABN). On the other hand, breast muscles without white striations or harden areas and hemorrhages have been classified as “macroscopically normal” (NORM). Subsequently, each FG sample (10/sampling time) has been analyzed by means of Hematoxylin and Eosin (H&E) staining to provide a more precise and accurate classification of the breasts based not only on the macroscopic features but also on the histological traits of the samples (e.g., increased deposition of fat and connective tissue, inflammatory cells infiltration and presence of necrotic fibers to confirm the classification as ABN samples). Then, for each sampling time, 5 FG (based on the macroscopic and microscopic examination) and 5 MG samples have been selected and considered for further analyses.

The muscles selected for gene expression analysis were snap-frozen in liquid nitrogen and transported on dry ice to the laboratory of Food Biotechnology Research Team (BIOTEC, Pathum Thani, Thailand). Upon arrival, the frozen samples were stored at -80°C until RNA isolation and gene expression analysis.

### RNA isolation and cDNA synthesis

Total RNA was isolated from *P*. *major* muscles using TriReagent (Molecular Research Center, Inc., Cincinnati, OH, USA) according to the manufacturer’s recommended protocol. DNA was removed by incubating the isolated RNA with DNase I (Thermo Scientific, Inc., Rockford, IL, USA). Total RNA samples were subsequently purified using a GeneJet RNA Cleanup and Concentration Micro kit (Thermo Scientific, Inc.). Total RNA concentration and quality were quantified using a Nanodrop spectrophotometer (model 2000, Thermo Scientific, Inc., Wilmington, DE, USA), and its integrity was further determined using an agarose gel electrophoresis. Total RNA was then proceeded to cDNA synthesis using an oligo(dT) as a primer and an ImProm-IITM Reverse Transcription System kit (Promega Corporation, Madison, WI, USA). The amount of the synthesized cDNA was determined using a Nanodrop spectrophotometer.

### Primers

A total of 33 genes were selected as our target gene markers based on their biological functions. The selected genes could be classified into five major groups, including muscle growth, hypoxia and oxidative stress response, AMPK and AMPK-related kinases, carbohydrate metabolisms, and satellite cells activities and fibro-adipogenic progenitors under muscle regeneration (Table 1).

**Table 1:** Target genes.

Molecular activity	Genes
Muscle growth	insulin-like growth factor 1 (*IGF1*), myogenic differentiation 1 (*MYOD1*), myogenic factor 5 (*MYF5*), myostatin (*MSTN)*
Hypoxia and oxidative stress response	hypoxia-inducible factor 1 A (*HIF1A*), glutathione S-transferase mu 2 (*GSTM2*), mitogen-activated protein (MAP) kinase interacting serine/threonine kinase 1 (*MKNK1*), cytosolic superoxide dismutase 1 (*SOD1*), mitochondrial superoxide dismutase 2 (*SOD2*), extracellular superoxide dismutase 3 (*SOD3*)
Carbohydrate metabolism	lactate dehydrogenase isoform A (*LDHA*), lactate dehydrogenase isoform B (*LDHB*), 6-phosphofructo-2-kinase/fructose2,6-biphosphatase 4 (*PFKFB4*)
AMPK and AMPK-related kinases	AMPKɑ1 isoform (*PRKAA1*), AMPKɑ2 isoform (*PRKAA2*), AMPKβ1 isoform (*PRKAB1*), AMPKβ2 (*PRKAB2*), AMPKγ1 isoform (*PRKAG1*), AMPKγ2 isoform (*PRKAG2*), AMPKγ3 isoform (*PRKAG3*), Ca^2+/^calmodulin dependent protein kinase kinase beta (*CAMKK2*), liver kinase 1 (*LKB1*), mechanistic target of rapamycin (*mTOR*), novel kinase family 1 (*NUAK1*)
Satellite cells activities and fibro-adipogenic progenitors under muscle regeneration	annexin A2 (*ANXA2*), desmin (*DES*), vimentin (*VIM*), transforming growth factor-beta 1 (*TGFB1*), platelet-derived growth factor alpha subunit (*PDGFA*), platelet-derived growth factor receptor alpha subunit (*PDGFRA*), matrix metalloproteinase-14 (*MMP14*), bone morphogenetic protein 1 (*BMP1*), lipopolysaccharide-induced tumor necrosis factor-alpha factor (*LITAF*).

Reference sequences obtained from the National Center for Biotechnology Information (NCBI) were used as the template for primer design. All primers were designed using Primer-BLAST (https://www.ncbi.nlm.nih.gov/tools/primer-blast/) and are listed in the S1 Table. Amplicon sizes were limited up to 200 bp. OligoAnalyzer (https://www.idtdna.com/calc/analyzer) was used for secondary structure and dimer prediction. Primers that matched recommended criteria (GC content 40–60%, melting temperature 50–65°C, ΔG > -5) were synthesized.

### Droplet digital polymerase chain reaction

Each droplet digital polymerase chain reaction (ddPCR) mixture comprised 1X EvaGreen supermix (Bio-Rad Laboratories, Inc., Hercules, CA, USA), 0.25 μM of each forward and reverse primers, and cDNA template at the optimal amount as specified in S1 Table and nuclease-free water was added to make up the total volume of 20 μL. A no template control was included in every run by replacing the cDNA template with an equal volume of nuclease-free water. The reaction mixture was loaded into QX100™ droplet generator (Bio-Rad Laboratories, Inc.) according to the instruction to generate water-in-oil droplet emulsion. Subsequently, the water-in-oil emulsion was manually transferred to a 96-well plate. The ddPCR reaction was performed in a T100™ thermal cycler (Bio-Rad Laboratories, Inc.) with the temperature profile set as follows; denaturation at 95°C for 5 min, annealing and extension at 95°C for 30 s followed by decreasing temperature to optimal annealing temperature as specified in S1 Table for 1 min for 40 cycles; droplet stabilization at 4°C for 5 min; and 90°C for 5 min. The temperature ramp rate of all steps was set at 2.5°C/min. Following the amplification, the fluorescent signal intensity of the droplets was measured using a QX200 droplet reader (Bio-Rad Laboratories, Inc.). Positive and negative droplets were counted and converted to the initial concentration (copies number per 20μL reaction) of the targets using a QuantaSoft droplet reader software (Bio-Rad Laboratories, Inc.). Absolute abundance was further calculated to copies per nanogram template by dividing the copy number per 20μL reaction by the amount of cDNA added into the reaction.

### Statistical analysis

Statistical analysis was performed using the R package version 3.2.1. Differences in means among age groups of each chicken strain were assessed using one-way analysis of variance, by setting the sampling time as the main effect. Data groups with significant differences in means between age groups within each strain (p<0.05) were separated using Duncan’s new multiple range test. The effects of the strain on absolute gene expression were determined between FG and MG groups at the same age using a non-parametric *t-*test (Mann-Whitney U-test) chosen due to the small number of replicates per line and developmental stage which does not result in a normal distribution of the results. The significance level for all statistical analyses was set at α = 0.05.

## Results

Concerning muscle classification, all FG samples collected at 7d, 14d, and 21d of age were macroscopically normal (NORM), whilst all samples collected in the last three sampling times (28d, 35d and 42d) were characterized by the presence of muscular abnormalities (ABN). With regard to MG broilers, all samples have been classified as NORM.

### Genes associated with muscle growth

**Fig 1:**
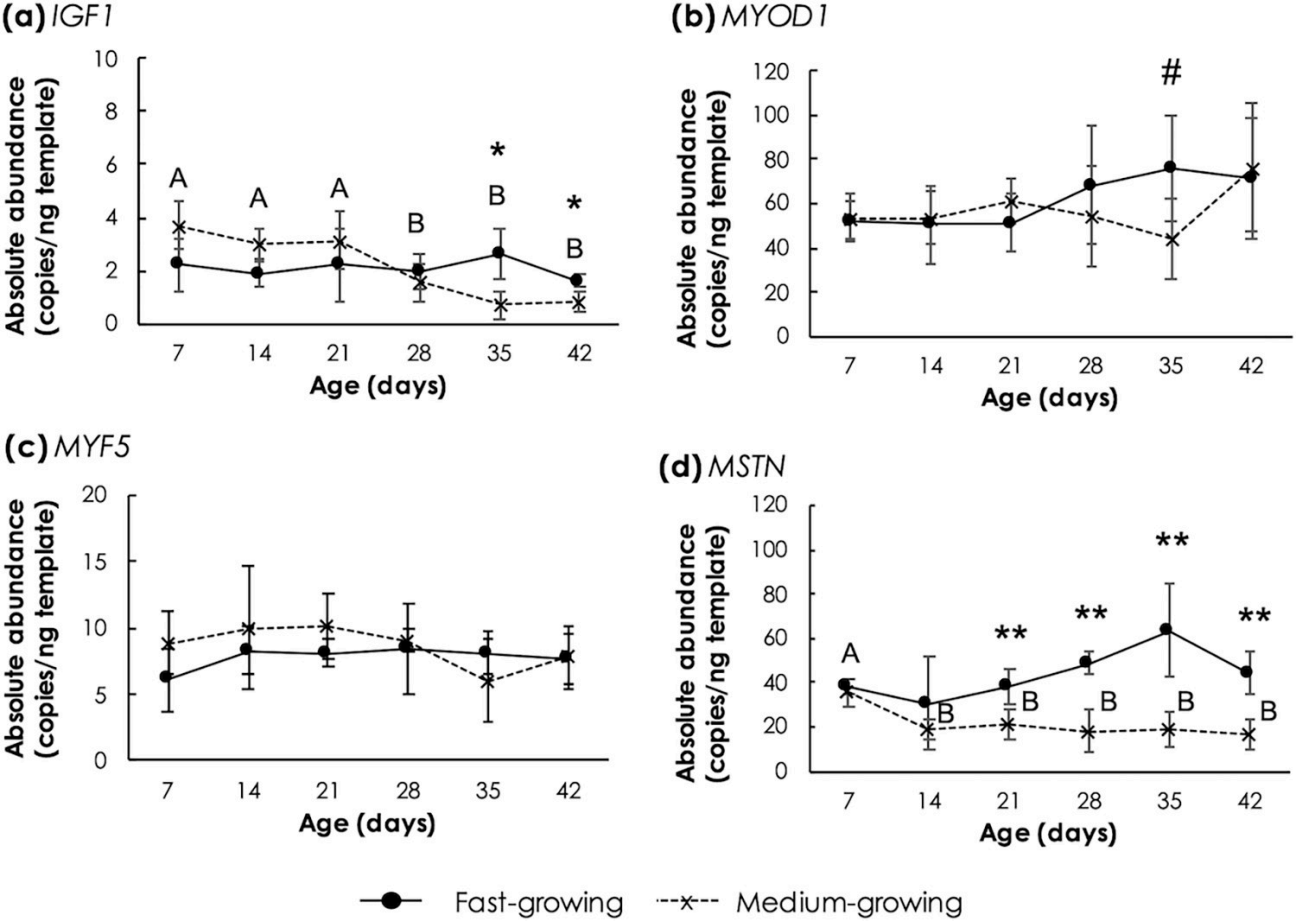
Absolute transcript abundance of genes involved in muscle growth in *Pectoralis major* muscle of fast- and medium-growing chickens at different ages. The genes include (a) insulin-like growth factor 1 (*IGF1*), (b) myogenic differentiation 1 (*MYOD1*), (c) myogenic factor 5 (*MYF5*) and (d) myostatin (*MSTN*). Markers and error bars depict mean value and standard deviation (n = 5). Upper case letters indicate significant differences among medium-growing chickens. Asterisks and sharps indicate significant differences between the two strains at the same age. ^#^p<0.1, *p<0.05, **p<0.01.

Absolute expression levels of genes associated with muscle growth are shown in Fig 1. Differential expression pattern of the tested genes between FG and MG was observed. Focusing on FG, as the age increased, no significant changes were observed for *IGF1*, *MSTN*, *MYF5* and *MYOD1* (p≥0.05). As for MG, *IGF1* abundance at the age of 28d, 35d and 42d were lower than (p<0.05) those at the age below 21d. In addition, *MSTN* level in the MG group was at the greatest level at the age of 7d (p<0.05). The expression of *MSTN* remained unchanged afterwards. Comparing the strains, FG samples exhibited greater abundances (p<0.05) of *IGF1* (on 35d and 42d) and *MSTN* (on 21d to 42d) than those of MG at later developmental stages.

### Genes involved in hypoxia and oxidative stress response

**Fig 2:**
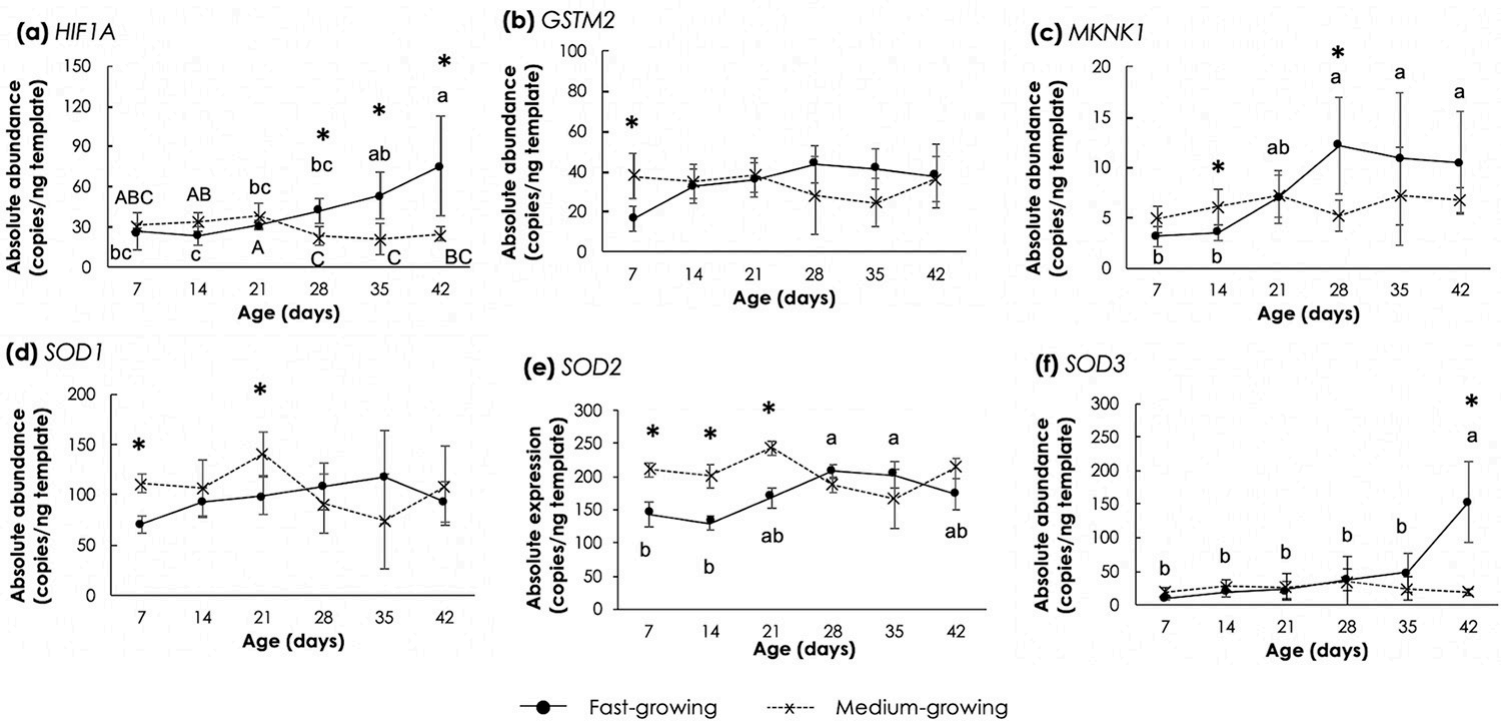
Absolute transcript abundance of genes responsible for hypoxia and oxidative stress response in *Pectoralis major* muscle of fast- and medium-growing chickens at different ages. The genes include (a) hypoxia-inducible factor 1 A (*HIF1A*), (b) glutathione S-transferase mu 2 (*GSTM2*), (c) mitogen-activated protein (MAP) kinase interacting serine/threonine kinase 1 (*MKNK1*), (d) cytosolic superoxide dismutase 1 (*SOD1*), (e) mitochondrial superoxide dismutase 2 (*SOD2*), and (f) extracellular superoxide dismutase 3 (*SOD3*). Markers and error bars depict mean and standard deviation (n = 5). Lower case letters indicate significant differences among fast-growing chickens. Upper case letters indicate significant differences among medium-growing chickens. Asterisks indicate significant differences between the two chicken strains at the same age. *p<0.05, **p<0.01, ***p<0.0001.

In Fig 2, the absolute expression levels of genes associated with the cellular stress response induced by low oxygen availability and oxidative stress are shown. Considering *HIF1A* (Fig 2A), as the age increased, *HIF1A* abundance in FG samples was increased (p<0.05) whereas, in MG, the expression of this gene was steady in the first 21d. Then, it slightly reduced (p<0.05) when the birds were at the age of 28d and remained constant thereafter. Comparing the chicken strains, *HIF1A* was expressed to a greater extent (p<0.05) in FG between 28d to 42d when compared to MG. As for the genes encoding for key antioxidant enzymes, i.e., *GSTM2*, *MKNK1*, *SOD1*, *SOD2* and *SOD3* (Fig 2B–2F), no significant changes were observed during growth in MG genotype. In FG, on the other hand, *MKNK1* and *SOD2* abundance in FG slightly increased up to 28d, and then it remained unchanged (Fig 2C and 2E). As for *SOD3*, its expression level achieved the highest value in FG at 42d (Fig 2F). The current results suggested that *P*. *major* of the FG might be under an oxidative stress particularly at the age of 28d onwards.

### Genes involved in carbohydrate metabolism

**Fig 3:**
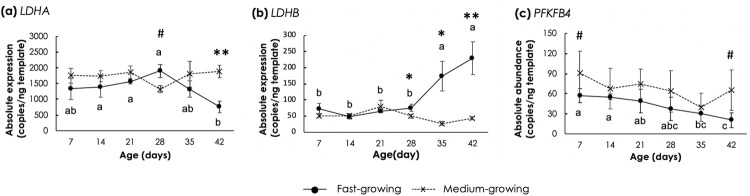
Absolute transcript abundance of genes involved in carbohydrate metabolism in *Pectoralis major* muscle of fast- and medium-growing chickens at different ages. The genes include (a) lactate dehydrogenase isoform A (*LDHA*), (b) lactate dehydrogenase isoform B (*LDHB*), and (c) 6-phosphofructo-2-kinase/fructose2,6-biphosphatase 4 (*PFKFB4*). Markers and error bars depict mean and standard deviation (n = 5). Lower case letters indicate significant differences among fast-growing chickens. Asterisks and sharp signs indicate significant differences between the two strains at the same age. ^#^p<0.1, *p<0.05, **p<0.01.

Differential expression patterns of gene involved in carbohydrate metabolism were detected between FG and MG chickens (Fig 3). In FG, *LDHA* transcript level was steady at the early age and tended to decrease (p<0.05) as the birds reached 42d of age. A decrease in transcript abundance combined with the increasing age was also found for *PFKFB4*; expression of *PFKFB4* in FG appeared at lower extent than that of MG. On the other hand, *LDHB* levels increased with age in FG birds.

### Genes encoding AMPK isoforms and AMPK-related kinases

**Fig 4:**
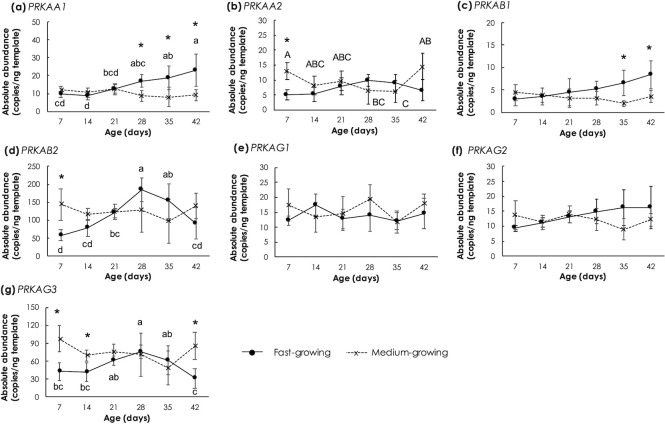
Absolute transcript abundance of genes encoding different isoforms of 5’-adenosine monophosphate-activated protein kinase (AMPK) in *Pectoralis major* muscle of fast- and medium-growing chickens at different ages. The AMPK isoforms include (a) ɑ1 isoform (*PRKAA1*), (b) ɑ2 isoform (*PRKAA2*), (c) β1 isoform (*PRKAB1*), (d) β2 (*PRKAB2*), (e) γ1 isoform (*PRKAG1*), (f) γ2 isoform (*PRKAG2*), and (g) γ3 isoform (*PRKAG3*). Markers and error bars depict mean and standard deviation (n = 5). Lower case letters indicate significant differences among fast-growing chickens. Upper case letters indicate significant differences among medium-growing chickens. Asterisks indicate significant differences between the two strains at the same age. *p<0.05, **p<0.01.

In this study, the expression of seven genes encoding each AMPK subunits and isoforms were examined in each chicken strain (Fig 4). In FG, absolute abundance of *PRKAA1* (Fig 4A) increased (p<0.05) as the age increased while its expression remained unchanged in MG. In detail, from 28d to 42d, *PRKAA1* was expressed to a greater extent in comparison with those of MG (p<0.05). On the other hand, expression of *PRKAA2* in FG was likely steady and, in absolute terms, its expression at 7d was significantly lower than that assessed for MG (Fig 4B). Expression of *PRKAB2* and *PRKAG3* in FG showed similar trend, since both their abundances gradually increased up to the age of 28d and reduced afterwards. No significant changes of *PRKAB1*, *PRKAG1* and *PRKAG2* expression during growing were observed (p≥0.05) which might be explained by the fact that those isoforms are not the main isoforms in chicken skeletal muscle [36]. In addition, differential gene expression patterns of the three skeletal muscle specific isoforms, i.e., *PRKAA2*, *PRKAB2* and *PRKAG3*, between FG and MG may reflect the different cellular energy status and metabolic activities during growth between the two strains.

**Fig 5:**
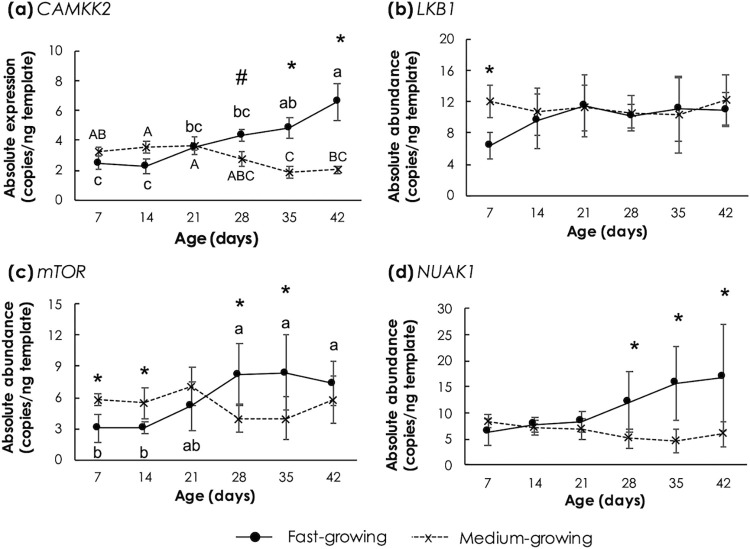
Absolute transcript abundance of genes encoding upstream and downstream kinases of 5’-adenosine monophosphate-activated protein kinase (AMPK) in *Pectoralis major* muscle of fast- and medium-growing chickens at different ages. The genes include (a) Ca^2+/^calmodulin dependent protein kinase kinase beta (*CAMKK2*), (b) liver kinase 1 (*LKB1*), (c) mechanistic target of rapamycin (*mTOR*), and (d) novel kinase family 1 (*NUAK1*). Markers and error bars depict mean and standard deviation (n = 5). Lower case letters indicate significant differences among fast-growing chickens. Upper-case letters indicate significant differences among medium-growing chickens. Asterisks and sharp sign indicate a significant difference between the two strains at the same age. ^#^p<0.1, *p<0.05, **p<0.01.

Expressions of genes encoding AMPK upstream (*CAMKK2* and *LKB1*) and downstream (*mTOR*) kinases along with AMPK-related kinase (*NUAK1*) were also investigated (Fig 5). As the age increased, *CAMKK2* abundance was increased in FG (Fig 5A) whereas an opposite trend was observed in MG. As for *LKB1*, no significant effects of age were found for either FG or MG (Fig 5B). The expression levels of *mTOR* (Fig 5C) in FG were increased from 7d to 28d and remained unchanged afterwards. No significant changes in *mTOR* levels were found in MG. Comparing FG and MG samples, *mTOR* was higher expressed (p<0.05) in MG than FG at the age of 7 and 14 days; however, this trend went into the opposite direction at the age of 21d and 35d. No significance of *NUAK1* (Fig 5D) regarding developmental stage was detected either in FG or MG (p ≥0.05) although its levels in FG tended to be increased at the later growing phase. The lack of significant change of the *NUAK1* expression in the FG during the age of 28d to 42d was due mainly to the large variation among the birds. However, a greater *NUAK1* abundance in FG than that in MG was detected at the age of 28d to 42d (p<0.05).

### Genes associated with activities of satellite cells and fibro-adipogenic progenitors (FAPs) during muscle regeneration

**Fig 6:**
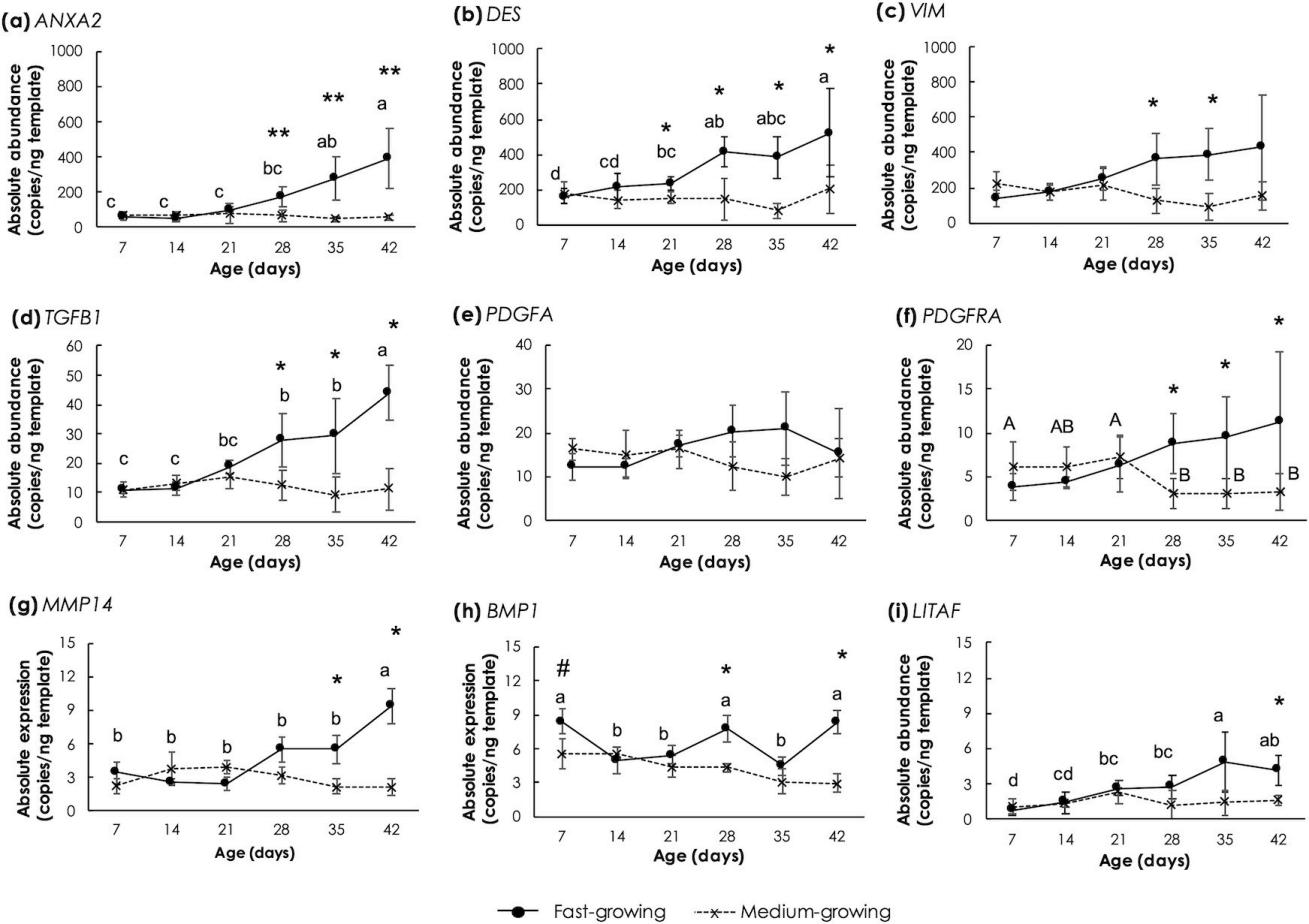
Absolute transcript abundance of genes associated with activities of satellite cells and fibro-adipogenic progenitors in *Pectoralis major* muscle of fast- and medium-growing chickens at different ages. The genes include (a) annexin A2 (*ANXA2*), (b) desmin (*DES*), (c) vimentin (*VIM*), (d) transforming growth factor beta 1 (*TGFB1*), (e) platelet-derived growth factor-alpha subunit (*PDGFA*), (f) platelet-derived growth factor receptor alpha subunit (*PDGFRA*), (g) Matrix metalloproteinase-14 (*MMP14*), (h) bone morphogenetic protein 1 (*BMP1*) and (i) lipopolysaccharide-induced tumor necrosis factor-alpha factor (*LITAF*). Markers and error bars depict mean and standard deviation (n = 5). Lower case letters indicate significant differences among fast-growing chickens. Upper case letters indicate significant differences among medium-growing chickens. Asterisks indicate significant differences between the two strains at the same age. *p<0.05, **p<0.01, ***p<0.0001.

Absolute transcript abundances of genes associated with satellite cells activity and fibro- adipogenic progenitors in breast muscle of FG and MG chickens are shown in Fig 6. Focusing on FG, *ANXA2*, *DES*, *TGFB1* and *LITAF*, their levels were increased (p<0.05) as the bird age increased. The greatest *MMP14* abundance (p<0.05) in the FG samples was detected at 42d. Expression of *BMP1* exhibited fluctuating patterns where *BMP1* transcripts were increased (p<0.05) at 7d, 28d, and 42d. With regard to MG samples, no significant changes in the tested genes were observed (p ≥ 0.05) with the only exception of a decrease in the *PDGFRA* expression level in the last three sampling times, if compared with the earlier ages.

## Discussion

Artificial selection has resulted in a massive improvement in growth performance and meat yield among fast-growing commercial broilers. However, the FG birds are more susceptible to a variety of stresses, including growth-related myopathies. The objective of this study was to monitor the changes in transcript abundance of the genes during post-hatch developmental stage of FG and MG chickens. The genes of interest were previously identified to be differentially expressed between normal and WS/WB-affected *P*. *major* muscle of broilers. In this regard, an investigation of those genes during growth may advance our understanding regarding the onset of growth-related myopathies in fast-growing birds. Initially, we anticipated to retrieve a few normal samples at the later age of FG and abnormal muscles of MG to be compared with their counterparts. However, since all FG samples collected in the last three sampling times (28d, 35d and 42d) were characterized by the presence of muscular abnormalities (ABN), whilst all MG samples have been classified as NORM, these results seem to support the association between breeding selection for fast growth rate and muscular abnormalities.

Given the importance of paracrine regulators in regulating muscle growth, changes of *IGF1*, *MSTN* and *MYOD1* were expected during growth and between FG and MG samples. Up-regulations of *IGF1* and *MYOD1* in breast muscle were previously observed in association with genetic selection for growth in turkeys [37], for high breast yield in chickens [38, 39] and for high feed efficiency in pedigree male broilers [40]. On the other hand, Kim et al. [41] reported no differential expression of *MSTN* in breast muscle after hatching between Japanese quails selected for low body weight and control line. The study of Xiao et al. [42] addressed that despite the different growth rate, transcript abundance of *IGF1* along with *MYOD1*, *MYF5* and *MSTN* in breast muscle did not differ between fast-growing and medium-growing broilers. In addition, Praud et al. [9] reported no changes of *MYOD1* and *MYF5* transcript levels in breast muscle among slow-growing chickens and broilers exhibited either normal characteristics, WS, WB, or WS/WB abnormalities.

Research has shown that in high-breast-yield chickens, breast thickening due to the muscle growth may lead to a decreased vascularization [43, 44], resulting in an accumulation of metabolic wastes (e.g., ROS) within the muscle. The events of hypoxia and oxidative stress have been speculated as the main condition triggering the onset of muscle damage that leads to growth-related myopathies in commercial broilers [10]. Antioxidant enzymes are, in part, responsible for maintaining low levels of these oxidant metabolites in the tissues. In this study, an increased expression of *HIF1A* currently identified in FG muscle was consistently observed in breast muscle of broilers exhibiting WS and WB [6], thus supporting the hypothesis of the development of hypoxic conditions in FG muscles. Among the enzymatic defense mechanisms against ROS, overexpression of *SOD2*, encoding the SOD2 isoform localized in mitochondria, in mice leads to enhanced mitochondrial function [45], preserving differentiation potential of myoblast [46], and protection from various oxidant stressors including reperfusion injury [47]. On the other hand, SOD3 is the only isoform secreted in the extracellular space; hence, it plays a critical role in preventing cell and tissue damage initiated by extracellularly produced ROS [48]. Okutsu et al. [49] addressed that apart from its role in scavenging extracellular superoxide, SOD3 could be internalized by the cells of glycolytic fibers and provided protective effects against extracellular ROS-mediated catabolic wasting. Laatikainen et al. [50] demonstrated that overexpressing *SOD3* in the hindlimb ischemic rats decreased apoptotic signaling and attenuated ischemic tissue injury. An increase in *SOD2* at the age of 28 days and *SOD3* at 42 days accompanied by the rise in *HIF1A* expression in FG chickens supports the existence of severe oxidative stress occurring in these muscles. More generally, this condition might be considered an adaptive mechanism to alleviate the muscle damage linked to the myopathies.

Differences in growth rate due to an intensive breeding selection for production efficiency have led to considerable differences in mechanisms of growth and development, hence in chicken metabolism [51, 52]. Selection for high breast yield in FG chickens partitions more nutrients toward the breast muscle, which may be particularly susceptible to metabolic perturbations due to its primary composition of type IIB glycolytic muscle fibers [53]. Such differences are still detected in this study in which FG and MG were compared. Interestingly, *LDHA* and *LDHB* showed an opposite direction of transcriptional changes in the FG muscle during the stage from 28d to 42d (Fig 3A and 3B). Such differential patterns of *LDHA* and *LDHB* expression were observed in highly proliferated cells, such as colorectal cancer cells [54] and lung cancer cells [55]. Suppression of *LDHA* expression in breast cancer cells induced cellular ROS accumulation with alteration of mitochondrial function, morphology, and metabolisms [56], thus resembling the pathological mitochondrial clearance observed in the breast muscle of chickens affected with severe WB abnormality [57]. In addition, LDHB has been shown to control basal autophagy of oxidative cancer cells [58]. Given that all FG chickens collected from 28d to 42d exhibited myopathic lesions, decreased levels of *LDHA* and an increased *LDHB* along with the changes in expression of genes related with oxidative stress response in the FG samples might suggest an induction of autophagy during the age of 28d to 42d in response to nutrient deprivation due to limited vascularization [59].

Since AMPK is widely known as an important kinase acting as fuel cellular energy sensor, changes in its expression are of particular interest to draw a more complete scenario of the molecular cascade in the FG and MG birds during growing. In this study, no significant changes of *PRKAB1*, *PRKAG1* and *PRKAG2* expression during growing were observed either in FG or MG samples. These results might be explained by the fact that those isoforms are not the main isoforms in chicken skeletal muscle [36].

Considering the expression patterns of those AMPK upstream kinases and *PRKAA1*, the increased *PRKAA1* in FG during growth observed in this study was consistent with an association between the up-regulated activity of AMPK with age in fast-twitch skeletal muscle [60]. Elevated expression of *PRKAA1*, primarily activated by CAMKKβ, was shown to be involved in limiting muscle hypertrophy [61, 62] through inhibition of the mTOR signaling pathway. In addition, using HepG2 cell lines, Suzuki et al. [63] demonstrated that, activated NUAK1, a serine/threonine kinase belonging to the AMPK-related kinase family, was essential for Akt-induced cell survival signaling during glucose deprivation and may influence AMPKɑ1 to induce cell tolerance to glucose starvation under hypoxia. NUAK1 is also required for Ca^2+^-dependent AMPK activity in absence of LKB1 and, together with AMPK, inhibition of mTOR complex 1 [64]. Taken together, up-regulation of *PRKAA1* and *NUAK1* identified in the FG samples suggested metabolic alteration of the cells due to hypoxia-induced glucose starvation while such event was not observed in the current MG samples during the growth [20, 62, 63].

LKB1 and CAMKKβ are widely known as the two main upstream kinases of AMPK. Unlike LKB1, AMPK activation by CaMKKβ does not require an alteration of ATP to AMP ratio but rather occurs in response to an increased intracellular Ca^2+^ concentration [65]. Today, the actual role of CAMKKβ in skeletal muscle is still under investigation. Up-regulated *CAMKK2* in the FG might partly be induced by an intracellular Ca^2+^ overload following muscle damage [66] consistently reported in breast muscles affected by WS and WB abnormality [12, 67, 68].

In this study, an increased *mTOR* abundance was detected in FG muscles between 28d and 42d (Fig 5C). In general, mTOR and AMPK function in an antagonistic manner to regulate the catabolic and anabolic balance of protein, energy homeostasis, and muscle hypertrophy [62, 69, 70]. However, a previous RNA-seq found up-regulation of both *PRKAA1* and *mTOR* in breast muscle of Pedigree male broilers with high feed efficiency (FE) compared to those with low FE phenotype [71]. Whether the enrichment of both *mTOR* and *PRKAA1* abundance in the previous study was due to inherent differences or to signal transduction mechanisms was still unclear. Nevertheless, Piekarski-Welsher et al. [71] speculated on competing signals between activation of the autophagy pathway through AMPKα 1 and inhibition of autophagy via mTOR in the muscle of high FE chickens. In this regard, it is reasonable to hypothesize, based upon our current findings, that similar competing signals between those two signaling pathways for regulating autophagy also occurred in breast muscle of FG samples, the modern broilers selected for feed efficiency, at the age of 28d to 42d. Further studies, particularly at protein levels, are required to gain more comprehension on this aspect.

To this point, differential gene expression patterns between FG and MG highlighted in this study pointed out the occurrence of an extensive muscle injury in FG breast muscle as the age of 28d onwards. This statement was supported by up-regulated *DES* and *VIM* in FG relative to those of MG at the later developmental stages. The two genes, also known as the markers for muscle regenerative process, encode intermediate filaments proteins contributing to the sarcomere integrity [72]. Consistent with our results, Soglia et al. [14] recently reported that the breast muscles of a slow-growing strain expressed lower *DES* (p = 0.08) and *VIM* (p = 0.10) if compared with unaffected broilers. Upon muscle injury, TNF-α and TGF-β are recruited to regulate muscle repair program. For the gene encoding chicken TNF-α, because the gene contains high GC content and long GC-rich stretches [73], it was not able to amplify by ddPCR. Therefore, instead of a direct investigation on the gene encoding TNF- α, absolute abundance of *LITAF*, a transcription factor of mammalian TNF- α, was examined in this study. Up-regulation of *TGFB1* and *LITAF* indicate an ongoing muscle injury in the breast muscle of FG birds during growing [33]. Additionally, expression of TGF-β is positively correlated with differentiation of FAPs into fibrogenic cells, leading to fibrosis [33, 74–76].

We also identified high absolute transcript abundances of *PDGFRA*, encoding a surface protein receptor located on FAP cells and considered as FAPs marker [77]. Similarly, *MMP14* and *BMP1*, encoding FAPs-expressed TGF- β activating proteases [76], showed high transcript levels in FG muscle at 42d, suggesting increased fibrotic activities of FAPs in the FG breast muscle. In the case of regular muscle regeneration, FAPs reciprocally regulated by satellite cells in the early phase are activated and differentiated into connective tissues to provide a transient source of pro-differentiation for proliferating satellite cells [30]. In the late phase, FAPs undergo TNF-α-induced apoptosis and return to the basal level [28]. However, in chronic inflammatory conditions, expression of *TGFB1* was markedly increased [30]. Juban et al. [76] demonstrated that under a muscle dystrophic environment, pro-inflammatory macrophages, instead of inhibiting FAPs proliferation and differentiation, secreted a high amount of latent TGF-β which was further activated by proteolytic enzymes, particularly MMP14 and BMP1, produced by FAPs. The active TGF-β acted in turn on FAPs to promote fibrosis. Increases in transcript abundance of *TGFB1*, *MMP14*, *BMP1* and *PDGFRA* in *P*. *major* might reflect the shift of FAP activities towards fibrosis within the muscle of FG at the age of 28 onwards. However, further analyses are necessary to verify this hypothesis.

Increased *PDGFRA* abundance was also addressed in the studies of Pampouille et al. [35] and Praud et al. [9] in which no differences in *PDGFRA* abundance between slow-growing and unaffected fast-growing chickens were observed, whilst the expression of this gene was significantly increased in the presence of WB abnormality. In addition, transcript abundance of *ANXA2*, encoding a Ca^2+^-dependent membrane repair protein with diverse biological functions, was elevated in the FG muscle as FG aged. Accumulated ANXA2 at the injured sites has been shown to create a pro-adipogenic environment, leading to FAP accumulation and adipogenic replacement in the muscle [78]. Altogether, the current findings suggested that FAPs activity may have a role in determining FG muscle features.

Overall, the current findings underlined differential expression patterns of the genes involved in oxidative stress response, muscle growth, and carbohydrate metabolism between FG and MG during growth. Chronic muscle regeneration might be hypothesized in the FG muscle at the age of 28 days onwards. Under such condition, AMPK/CAMKKβ, mTOR and TGF-β signaling might play roles in inducing FAP activity towards fibrosis. It is worth noting that all FG samples from 28d to 42d exhibited abnormalities at macroscopic observation (i.e., white striations or harden areas and hemorrhages) and histological detection (e.g., increased deposition of fat and connective tissue, inflammatory cells infiltration and presence of necrotic fibers). The results support the hypothesis that fast growth rate may lead to the altered biological and molecular processes resulting in the myopathic lesions affecting fast-growing chickens. On the other hand, this exerted a limitation in our study as there was no normal samples to be compared with the affected ones. Further gene expression analysis comparing between normal and affected FG muscle samples is required to test the hypothesis.

## Conclusion

In conclusion, the differential expression of the target gene markers indicated molecular differences in muscle growth, metabolic shifts, stress response, and muscle repair, between FG and MG pectoral muscles during growth. Our results also suggest that the chronic inflammatory muscle injury in the FG muscles, especially at 28d post-hatch onwards, might have created a stress environment that triggered aberrant FAP activity, resulting in fibrosis in the FG *P*. *major* muscle.

## Supporting information

S1 Table Primers, optimal template concentration and annealing condition. (XLSX)

## References

[1] Zanetti MA, Tedesco DC, Schneider T, Teixeira STF, Daroit L, Pilotto F, et al. Economic losses associated with Wooden breast and White Striping in broielrs. Semin Agrar. 2018;39(2):887–891. doi: 10.5433/1679-0359.2018v39n2p887

[2] Velleman SG. Relationship of Skeletal Muscle Development and Growth to Breast Muscle Myopathies: A Review. AVIAN DISEASES. 2015;59(4):525–531. doi: 10.1637/11223-063015-Review.1 26629627

[3] Sihvo H-K, Airas N, Lindén J, and Puolanne E. Pectoral vessel density and early ultrastructural changes in broiler chicken wooden breast myopathy. J Comp Pathol. 2018;161:1–10. doi: 10.1016/j.jcpa.2018.04.002 30173852

[4] Petracci M, Soglia F, Madruga M, Carvalho L, Ida E, and Estévez M. Wooden-breast, white striping, and spaghetti meat: Causes, consequences and consumer perception of emerging broiler meat abnormalities. Compr Rev Food Sci Food Saf. 2019;18:565–583. doi: 10.1111/1541-4337.12431 33336940

[5] Zambonelli P, Zappaterra M, Soglia F, Petracci M, Sirri F, Cavani C, et al. Detection of differentially expressed genes in broiler pectoralis major muscle affected by White Striping–Wooden Breast myopathies. Poultry Science. 2016;95(12):2771–2785. doi: 10.3382/ps/pew268 27591279

[6] Malila Y, Thanatsang K, Arayamethakorn S, Uengwetwanit T, Srimarut Y, Petracci M, et al. Absolute expressions of hypoxia-inducible factor-1 alpha (HIF1A) transcript and the associated genes in chicken skeletal muscle with white striping and wooden breast myopathies. PLoS ONE. 2019;14(8):e0220904. doi: 10.1371/journal.pone.0220904 31393948 PMC6687142

[7] Abasht B, Mutryn MF, Michalek RD, and Lee WR. Oxidative stress and metabolic perturbations in wooden breast disorder in chickens. PLoS ONE. 2016;11(4):e0153750. doi: 10.1371/journal.pone.0153750 27097013 PMC4838225

[8] Baldi G, Soglia F, Mazzoni M, Sirri F, Canonico L, Babini E, et al. Implications of white striping and spaghetti meat abnormalities on meat quality and histological features in broilers. Animal. 2018;12(1):164–173. doi: 10.1017/S1751731117001069 28528595

[9] Praud C, Jimenez J, Pampouille E, Couroussé N, Godet E, Bihan-Duval LE, et al. Molecular Phenotyping of White Striping and Wooden Breast Myopathies in Chicken. Front Physiol. 2020;11(633). doi: 10.3389/fphys.2020.00633 32670085 PMC7328665

[10] Ayansola H, Liao C, Dong Y, Yu X, Zhang B, and Wang B. Prospect of early vascular tone and satellite cell modulations on white striping muscle myopathy. Poult Sci. 2021;100(3):100945. doi: 10.1016/j.psj.2020.12.042 33652536 PMC7936185

[11] Sihvo HK, Immonen K, and Puolanne E. Myodegeneration with fibrosis and regeneration in the pectoralis major muscle of broilers. Vet Pathol. 2014;51(3):619–623. doi: 10.1177/0300985813497488 23892375

[12] Soglia F, Mudalal S, Babini E, Di Nunzio M, Mazzoni M, Sirri F, et al. Histology, composition, and quality traits of chicken Pectoralis major muscle affected by wooden breast abnormality. Poult Sci. 2016;95(3):651–659. doi: 10.3382/ps/pev353 26706363

[13] Baldi G, Yen C-N, Daughtry MR, Bodmer J, Bowker BC, Zhuang H, et al. Exploring the Factors Contributing to the High Ultimate pH of Broiler Pectoralis Major Muscles Affected by Wooden Breast Condition. Front Physiol. 2020;11:343. doi: 10.3389/fphys.2020.00343 32457639 PMC7227419

[14] Soglia F, Mazzoni M, Zappaterra M, Nunzio MD, Babini E, Bordini M, et al. Distribution and expression of vimentin and desmin in broiler pectoralis major affected by the growth-related muscular abnormalities. Front Physiol. 2020;10(1581). doi: 10.3389/fphys.2019.01581 32009982 PMC6978684

[15] Phillips CA, Reading BJ, Livingston M, Livingston K, and Ashwell CM. Evaluation via supervised machine learning of the broiler Pectoralis major and liver transcriptome in association with the muscle myopathy Wooden Breast. Front Physiol. 2020;11(101). doi: 10.3389/fphys.2020.00101 32158398 PMC7052112

[16] Velleman SG. Pectoralis Major (Breast) Muscle Extracellular Matrix Fibrillar Collagen Modifications Associated With the Wooden Breast Fibrotic Myopathy in Broilers. Front Physiol. 2020;11:461. doi: 10.3389/fphys.2020.00461 32457657 PMC7221152

[17] Bordini M, Zappaterra M, Soglia F, Petracci M, and Davoli R. Weighted gene co-expression network analysis identifies molecular pathways and hub genes involved in broiler White Striping and Wooden Breast myopathies. Scientific Reports. 2021;11(1):1776. doi: 10.1038/s41598-021-81303-7 33469097 PMC7815844

[18] Koh HJ, Brandauer J, and Goodyear LJ. LKB1 and AMPK and the regulation of skeletal muscle metabolism. Curr Opin Clin Nutr Metab Care. 2008;11(3):227–232. doi: 10.1097/MCO.0b013e3282fb7b76 18403917 PMC2887290

[19] Chan K-H, Lam KS-L, Cheng O-Y, Kwan JS-C, Ho PW-L, Cheng KK-Y, et al. Adiponectin is protective against oxidative stress induced cytotoxicity in amyloid-beta neurotoxicity. PLoS ONE. 2012;7(12):e52354. doi: 10.1371/journal.pone.0052354 23300647 PMC3531475

[20] Sanchez AM, Candau RB, Csibi A, Pagano AF, Raibon A, and Bernardi H. The role of AMP-activated protein kinase in the coordination of skeletal muscle turnover and energy homeostasis. Am J Physiol Cell Physiol. 2012;303(5):C475–485. doi: 10.1152/ajpcell.00125.2012 22700795

[21] Thomson DMM. The Role of AMPK in the Regulation of Skeletal Muscle Size, Hypertrophy, and Regeneration. Int J Mol Sci. 2018;19(10):3125. doi: 10.3390/ijms19103125 30314396 PMC6212977

[22] Rajakylä EK, Lehtimaki JI, Acheva A, Schaible N, Lappalainen P, Krishnan R, et al. ArticleAssembly of Peripheral Actomyosin Bundles inEpithelial Cells Is Dependent on the CaMKK2/AMPK Pathway. Cell Reports. 2020;30:4266–4280. doi: 10.1016/j.celrep.2020.02.096 32209483

[23] Yang W, and Hu P. Skeletal muscle regeneration is modulated by inflammation. J Orthop Translat. 2018;13:25–32. doi: 10.1016/j.jot.2018.01.002 29662788 PMC5892385

[24] Mounier R, Théret M, Arnold L, Cuvellier S, Bultot L, Göransson O, et al. AMPKa1 regulates macrophage skewing at the time of resolution of inflammation during skeletal muscle regeneration. Cell Metab. 2013;18(2):251–264. doi: 10.1016/j.cmet.2013.06.017 23931756

[25] Domingues-Faria C, Vasson M-P, Goncalves-Mendes N, Boirie Y, and Walrand S. Skeletal muscle regeneration and impact of aging and nutrition. Ageing Res Rev. 2016;26:22–36. doi: 10.1016/j.arr.2015.12.004 26690801

[26] Judson RN, Zhang RH, and Rossi FM. Tissue-resident mesenchymal stem/progenitor cells in skeletal muscle: collaborators or saboteurs?. FEBS J. 2013;280(17):4100–4108. doi: 10.1111/febs.12370 23763717 PMC4880469

[27] Theret M, Rossi FMV1, and Contreras O. Evolving Roles of Muscle-Resident Fibro-Adipogenic Progenitors in Health, Regeneration, Neuromuscular Disorders, and Aging. Front Physiol., 2021;12:673404. doi: 10.3389/fphys.2021.673404 33959042 PMC8093402

[28] Lemos DR, Babaeijandaghi F, Low M, Chang CK, Lee ST, Fiore D, et al. Nilotinib reduces muscle fibrosis in chronic muscle injury by promoting TNF-mediated apoptosis of fibro/adipogenic progenitors. Nat Med. 2015;21(7):786–794. doi: 10.1038/nm.3869 26053624

[29] Pagano AF, Arc-Chagnaud C, Brioche T, Chopard A, and Py G. Muscle Resting and TGF-β Inhibitor Treatment Prevent Fatty Infiltration Following Skeletal Muscle Injury. Cell Physiol Biochem. 2019;53(1):62–75. doi: 10.33594/000000121 31184447

[30] Contreras O, Rebolledo DL, Oyarzún JE, Olguín HC, and Brandan E. Connective tissue cells expressing fibro/adipogenic progenitor markers increase under chronic damage: relevance in fibroblast-myofibroblast differentiation and skeletal muscle fibrosis. Cell Tissue Res. 2016;364:647–660. doi: 10.1007/s00441-015-2343-0 26742767

[31] Giuliani G, Rosina M, and Reggio A. Signaling pathways regulating the fate of fibro/adipogenic progenitors (FAPs) in skeletal muscle regeneration and disease. FEBS J. 2021 Jun 18. doi: 10.1111/febs.16080 34143565

[32] Contreras O, Cruz-Soca M, Theret M, Soliman H, Tung LW, Groppa E, et al. Cross-talk between TGF-β and PDGFRα signaling pathways regulates the fate of stromal fibro-adipogenic progenitors. J Cell Sci. 2019;132(19):jcs232157. doi: 10.1242/jcs.232157 31434718

[33] Contreras O, Soliman H, Theret M, Rossi FMV, and Brandan E. TGF-β-driven downregulation of the transcription factor TCF7L2 affects Wnt/β-catenin signaling in PDGFRα+ fibroblasts. J Cell Sci. 2020;133(12):jcs242297. doi: 10.1242/jcs.242297 32434871

[34] Papah MB, Brannick EM, Schmidt CJ, and Abasht B. Gene expression profiling of the early pathogenesis of wooden breast disease in commercial broiler chickens using RNA-sequencing. PLoS One. 2018;13(12): e0207346. doi: 10.1371/journal.pone.0207346 30517117 PMC6281187

[35] Pampouille E, Hennequet-Antier C, Praud C, Juanchich A, Brionne A, Godet E, et al. Differential expression and co-expression gene network analyses reveal molecular mechanisms and candidate biomarkers involved in breast muscle myopathies in chicken. Sci Rep. 2019;9(1):14905. doi: 10.1038/s41598-019-51521-1 31624339 PMC6797748

[36] Proszkowiec-Weglarz M, Richards MP, Ramachandran R, and McMurtry JP. Characterization of the AMP-activated protein kinase pathway in chickens. Comp Biochem Physiol B Biochem. 2006;143(1):92–106. doi: 10.1016/j.cbpb.2005.10.009 16343965

[37] Liu C, McFarland DC, and Velleman SG. Effect of genetic selection on MyoD and myogenin expression in turkeys with different growth rates. Poult Sci. 2005;84(3):376–384. doi: 10.1093/ps/84.3.376 15782905

[38] Guernec A, Berri C, Chevalier B, Wacrenier-Cere N, Bihan-Duval EL, and Duclos MJ. Muscle development, insulin-like growth factor-I and myostatin mRNA levels in chickens selected for increased breast muscle yield. Growth Horm IGF Res. 2003;13(1):8–18. doi: 10.1016/s1096-6374(02)00136-3 12550077

[39] Jia J, Ahmed I, Liu L, Liu Y, Xu Z, Duan X, et al. Selection for growth rate and body size have altered the expression profiles of somatotropic axis genes in chickens. PLoS ONE. 2018;13(4):e0195378. doi: 10.1371/journal.pone.0195378 29630644 PMC5891002

[40] Lassiter K, Kong BC, Piekarski-Welsher A, Dridi S, and Bottje WG. Gene Expression Essential for Myostatin Signaling and Skeletal Muscle Development Is Associated With Divergent Feed Efficiency in Pedigree Male Broilers. Front Physiol. 2019;10(126). doi: 10.3389/fphys.2019.00126 30873041 PMC6401619

[41] Kim DH, Choi YM, Suh Y, Shin S, Lee J, Hwang S, et al. Research Note: Increased myostatin expression and decreased expression of myogenic regulatory factors in embryonic ages in a quail line with muscle hypoplasia. Poult Sci. 2021;100(4):100978. doi: 10.1016/j.psj.2021.01.001 33588344 PMC7896188

[42] Xiao Y, Wu C, Li K, Gui G, Zhang G, and Yang H. Association of growth rate with hormone levels and myogenic gene expression profile in broilers. J Anim Sci Biotechnol. 2017;8:43. doi: 10.1186/s40104-017-0170-8 28484596 PMC5420090

[43] Alnahhas N, Berri C, Chabault M, Chartrin P, Boulay M, Bourin MC, et al. Genetic parameters of white striping in relation to body weight, carcass composition, and meat quality traits in two broiler lines divergently selected for the ultimate pH of the pectoralis major muscle. BMC Genet. 2016;17(61). doi: 10.1186/s12863-016-0369-2 27094623 PMC4837622

[44] Kindlein L, Ferreira TZ, Driemeier D, Nascimento VP, Vieira SL, Moraes LE, et al. Occurrence and Severity of White Striping in Broilers Until 50d of Age Fed with High and Low-Energy Diets: Body Weight, Histopathological Changes and Meat Quality. J Vet Sci Technol. 2017;8(478). doi: 10.4172/2157-7579.1000478

[45] Silva JP, Shabalina IG, Dufour E, Petrovic N, Backlund EC, Hultenby K, et al. SOD2 over- expression: enhanced mitochondrial tolerance but absence of effect on UCP activity. EMBO J. 2005;24:4061–4070. doi: 10.1038/sj.emboj.7600866 16281056 PMC1356306

[46] Lee S, Remmen HV, and Csete M. Sod2 overexpression preserves myoblast mitochondrial mass and function, but not muscle mass with aging. Aging Cell. 2009;8(3):296–310. doi: 10.1111/j.1474-9726.2009.00477.x 19627269

[47] Suzuki K, Murtuza B, Sammut IA, Latif N, Jayakumar J, Smolenski RT, et al. Heat shock protein 72 enhances manganese superoxide dismutase activity during myocardial ischemia-reperfusion injury, associated with mitochon- drial protection and apoptosis reduction. Circulation. 2002;106:1270–1276. doi: 10.1161/01.cir.0000032880.55215.92 12354745

[48] Wang Y, Branicky R, Noë A, and Hekimi S. Superoxide dismutases: Dual roles in controlling ROS damage and regulating ROS signaling. J Cell Biol. 2018;217(6):1915–1928. doi: 10.1083/jcb.201708007 29669742 PMC5987716

[49] Okutsu M, Call JA, Lira VA, Zhang M, Donet JA, French BA, et al. Extracellular Superoxide Dismutase Ameliorates Skeletal Muscle Abnormalities, Cachexia, and Exercise Intolerance in Mice with Congestive Heart Failure. Circ Heart Fail. 2014;3:519–530. doi: 10.1161/CIRCHEARTFAILURE.113.000841 24523418 PMC4080303

[50] Laatikainen LE, Incoronato M, Castellone MD, Laurila JP, Santoro M, and Laukkanen MO. SOD3 Decreases Ischemic Injury Derived Apoptosis through Phosphorylation of Erk1/2, Akt, and FoxO3a. PLoS ONE. 2011;6(8):e24456. doi: 10.1371/journal.pone.0024456 21909393 PMC3164207

[51] Buzała M, Janicki B, and Czarnecki R. Consequences of different growth rates in broiler breeder and layer hens on embryogenesis, metabolism and metabolic rate: A review. Poult Sci. 2015;94(4):728–733. doi: 10.3382/ps/pev015 25691756

[52] Abasht B, Zhou N, Lee WR, Zhuo Z, and Peripolli E. The metabolic characteristics of susceptibility to wooden breast disease in chickens with high feed efficiency. Poultry Science. 2019;98:3246–3256. doi: 10.3382/ps/pez183 30995306

[53] Lake JA and Abasht B. Glucolipotoxicity: A Proposed Etiology for Wooden Breast and Related Myopathies in Commercial Broiler Chickens. Front Physiol. 2020;11(169). doi: 10.3389/fphys.2020.00169 32231585 PMC7083144

[54] Satoh K, Yachida S, Sugimoto M, Oshima M, Nakagawa T, Akamoto S, et al. Global metabolic reprogramming of colorectal cancer occurs at adenoma stage and is induced by MYC. P Natl A Sci. 2017;114(37):E7697–E7706. doi: 10.1073/pnas.1710366114 28847964 PMC5604037

[55] McCleland ML, Adler AS, Deming L, Cosino E, Lee L, Blackwood EM, et al. Lactate dehydrogenase B is required for the growth of KRAS-dependent lung adenocarcinomas. Clin Cancer Res. 2013;19(4):773–784. doi: 10.1158/1078-0432.CCR-12-2638 23224736

[56] Wang Z-Y, Loo TY, Shen J-G, Wang N, Wang D-M, Yang D-P, et al. LDH-A silencing suppresses breast cancer tumorigenicity through induction of oxidative stress mediated mitochondrial pathway apoptosis. Breast Cancer Res Tr. 2012;131(3):791–800. doi: 10.1007/s10549-011-1466-6 21452021

[57] Hosotani M, Kawasaki T, Hasegawa Y, Wakasa Y, Hoshino M, Takahashi N, et al. Physiological and pathological mitochondrial clearance is related to pectoralis major muscle pathogenesis in broilers with wooden breast syndrome. Front Physiol. 2020;11:579. doi: 10.3389/fphys.2020.00579 32612535 PMC7308532

[58] Brisson L, Bański P, Sboarina M, Dethier C, Danhier P, Fontenille M-J, et al. Lactate dehydrogenase B controls lysosome activity and autophagy in cancer. Cancer Cell. 2016;30(3):418–431. doi: 10.1016/j.ccell.2016.08.005 27622334

[59] Urbańska K, and Orzechowski A. Unappreciated role of LDHA and LDHB to control apoptosis and autophagy in tumor cells. Int J Mol Sci. 2019;20(9):2085. doi: 10.3390/ijms20092085 31035592 PMC6539221

[60] Gordon SE, Lake JA, Westerkamp CM, and Thomson DM. Does AMP-activated protein kinase negatively mediate aged fast-twitch skeletal muscle mass?. Exerc Sport Sci Rev. 2008;36(4):179–186. doi: 10.1097/JES.0b013e3181877e13 18815486 PMC2659413

[61] McGee SL, Mustard KJ, Hardie DG, and Baar K. Normal hypertrophy accompanied by phosphoryation and activation of AMP-activated protein kinase alpha1 following overload in LKB1 knockout mice. J Physiol. 2008;586(6):1731–1741. doi: 10.1113/jphysiol.2007.143685 18202101 PMC2327866

[62] Mounier R, Lantier L, Leclerc J, Sotiropoulos A, Pende M, Daegelen D, et al. Important role for AMPKalpha1 in limiting skeletal muscle cell hypertrophy. FASEB J. 2009;23(7):2264–2273. doi: 10.1096/fj.08-119057 19237506

[63] Suzuki A, Kusakai G, Kishimoto A, Lu J, Ogura T, Lavin MF, et al. Esumi H. Identification of a novel protein kinase mediating Akt survival signaling to the ATM protein. J Biol Chem. 2003;278(1):48–53. doi: 10.1074/jbc.M206025200 12409306

[64] Monteverde T, Tait-Mulder J, Hedley A, Knight JR, Sansom OJ, and Murphy DJ. Calcium signalling links MYC to NUAK1. Oncogene. 2018;37(8):982–992. doi: 10.1038/onc.2017.394 29106388 PMC5815498

[65] Green MF, Anderson KA, and Means AR. Characterization of the CaMKKβ-AMPK signaling complex. Cell Signal. 2011;23(12):2005–2012. doi: 10.1016/j.cellsig.2011.07.014 21807092 PMC3184326

[66] Soglia F, Petracci M, Davoli R, and Zappaterra M. A critical review of the mechanisms involved in the occurrence of growth-related abnormalities affecting broiler chicken breast muscles. Poult Sci. 2021;100(6):101180. doi: 10.1016/j.psj.2021.101180 33975044 PMC8131729

[67] Tasoniero G, Cullere M, Cecchinato M, Puolanne E, and Dalle ZA. Technological quality, mineral profile, and sensory attributes of broiler chicken breasts affected by White Striping and Wooden Breast myopathies. Poult Sci. 2016;95(11):2707–2714. doi: 10.3382/ps/pew215 27486252

[68] Thanatsang KV, Malila Y, Arayamethakorn S, Srimarut Y, Tatiyaborworntham N, Uengwetwanit T, et al. Nutritional Properties and Oxidative Indices of Broiler Breast Meat Affected by Wooden Breast Abnormality. Animals. 2020;10(12):2272. doi: 10.3390/ani10122272 33276466 PMC7759853

[69] Xu J, Ji J, and Yan X-H. Cross-talk between AMPK and mTOR in regulating energy balance. Crc Cr Rev Food Sci. 2012;52(5):373–381. doi: 10.1080/10408398.2010.500245 22369257

[70] Li Z, Miao Z, Ding L, Teng X, and Bao J. Energy metabolism disorder mediated ammonia gas-induced autophagy via AMPK/mTOR/ULK1-Beclin1 pathway in chicken livers. Ecotox Environ Safe. 2021;217:112219. doi: 10.1016/j.ecoenv.2021.112219 33853017

[71] Piekarski-Welsher A, Greene E, Lassiter K, Kong BC, Dridi S, and Bottje W. Enrichment of autophagy and proteosome pathways in breast muscle of feed efficient pedigree male broilers. Front Physiol. 2018;1342. doi: 10.3389/fphys.2018.01342 30416449 PMC6213487

[72] Marzuca-Nassr GN, Vitzel KF, Mancilla-Solorza E, and Márquez JL. Sarcomere Structure: The Importance of Desmin Protein in Muscle Atrophy. Int J Morphol. 2018;36(2):576–583. doi: 10.4067/S0717-95022018000200576

[73] Rohde F, Schusser B, Hron T, Farkašová H, Plachý J, Härtle S, et al. Characterization of Chicken Tumor Necrosis Factor-α, a Long Missed Cytokine in Birds. Front Immunol. 2018;9(605). doi: 10.3389/fimmu.2018.00605 29719531 PMC5913325

[74] Davies MR, Liu X, Lee L, Laron D, Ning AY, Kim HT, et al. TGF-β Small Molecule Inhibitor SB431542 Reduces Rotator Cuff Muscle Fibrosis and Fatty Infiltration By Promoting Fibro/Adipogenic Progenitor Apoptosis. PLoS ONE. 2016;11(5):e0155486. doi: 10.1371/journal.pone.0155486 27186977 PMC4871364

[75] Song Y, Yao S, Liu Y, Long L, Yang H, Li Q, et al. Expression levels of TGF-β1 and CTGF are associated with the severity of Duchenne muscular dystrophy. Exp Ther Med. 2017;13(4):1209–1214. doi: 10.3892/etm.2017.4105 28413459 PMC5377242

[76] Juban G, Saclier M, Yacoub-Youssef H, Kernou A, Arnold L, Boisson C, et al. AMPK Activation Regulates LTBP4-Dependent TGF-β1 Secretion by Pro-inflammatory Macrophages and Controls Fibrosis in Duchenne Muscular Dystrophy. Cell Rep. 2018;25(8):2163–2176.e6. doi: 10.1016/j.celrep.2018.10.077 30463013

[77] Arrighi N, Moratal C, Clément N, Giorgetti-Peraldi S, Peraldi P, Loubat A, et al. Characterization of adipocytes derived from fibro/adipogenic progenitors resident in human skeletal muscle. Cell Death Dis. 2015;6(4):e1733. doi: 10.1038/cddis.2015.79 25906156 PMC4650547

[78] Hogarth MW, Defour A, Lazarski C, Gallardo E, Manera JD, Partridge TA, et al. Fibroadipogenic progenitors are responsible for muscle loss in limb girdle muscular dystrophy 2B. Nat Commun. 2019;10(1):2430. doi: 10.1038/s41467-019-10438-z 31160583 PMC6547715

